# REM sleep latency as an independent risk for cardiovascular events in hemodialysis patients

**DOI:** 10.14814/phy2.14837

**Published:** 2021-05-15

**Authors:** Shigeichi Shoji, Masaaki Inaba, Koichiro Yoda, Hisanori Okazaki, Mio Toyokawa, Kyoko Norimine, Tomoyuki Yamakawa, Senji Okuno

**Affiliations:** ^1^ Department of Nephrology Shirasagi Hospital Osaka Japan; ^2^ Department of Metabolism, Endocrinology, Molecular Medicine and Nephrology Osaka City University Graduate School of Medicine Osaka Japan

**Keywords:** cardiovascular event, hemodialysis, REM sleep latency, sleep apnea, sleep quality

## Abstract

**Background:**

Clinical significance of objectively measured poor sleep quality (SQ) as a risk for cardiovascular disease (CVD) events is not well known in hemodialysis (HD) patients, independently of sleep‐related breathing disorders (SRBDs) and sleep‐related metabolic abnormality.

**Methods:**

The present study investigated baseline levels of objective sleep architecture together with obstructive sleep apnea (OSA) and central sleep apnea (CSA) using polysomnography in 88 HD study participants (M/F, 56/32; age 68.4 ± 9.3). Then, HD study participants were monitored for the occurrence of new‐onset CVD events with a median (range) follow‐up period of 33 (1–64) months.

**Results:**

Among various measures of SQ, log (REM sleep latency [REM‐SL]) (interval between sleep‐onset and the first REM period) alone correlated in negative manners with triglycerides and non‐HDL‐C in all study participants and with fasting plasma glucose and HbA1c in study participants with type‐2 diabetes mellitus. In the Kaplan–Meier analysis, HD study participants with shorter REM‐SL had a significantly higher rate of new‐onset CVD events than those with longer REM‐SL. Stepwise logistic regression analysis and multivariate Cox proportional hazard regression analysis identified shorter REM‐SL as an independent risk factor for the development of a new‐onset CVD events, independent of mean oxygen saturation, log (AHI+1), log (central AHI+1), diabetes mellitus, CVD history, systolic blood pressure, statins use, and non‐HDL‐C.

**Conclusions:**

The present study demonstrated that reduction of REM‐SL is independently associated with a higher rate of new‐onset of CVD events, independent of SRBDs (OSA and CSA) and diabetes mellitus, non‐HDL‐C in HD study participants, suggesting impaired SQ as a potential CVD risk factor, and thus a definite treatment target to protect against CVD specifically in HD study participants. REM‐SL might be a new risk factor of CVD events in HD patients with SRBDs.

## Graphical abstract

We found shorter REM sleep latency is an independent risk factor for the development of a new‐onset cardiovascular disease even in hemodialysis patients. Kaplan–Meier CVD Event‐free survival curves with REM sleep latency above and below the group median.

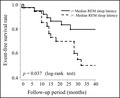

## 1 INTRODUCTION

Since aging and chronic kidney disease are both risk factors for various forms of sleep‐related disorders, including poor sleep quality (SQ), obstructive sleep apnea (OSA), central sleep apnea (CSA), restless legs syndrome, and periodic limb movement disorder (Carskadon & Dement, 1985; Rod et al., 2011; Tang et al., 2009), their prevalence becomes higher with the increasing numbers of elderly patients witht type 2 diabetes mellitus (T2DM) (Skomro et al., 2001). We recently reported that poor SQ, based on short REM sleep latency (REM‐SL) determined with electroencephalography, is independently associated with impaired lipid and glucose metabolisms in T2DM. leading to arterial wall stiffening in those patients (Yoda et al., 2015). However, it was reported that high prevalence of sleep‐related breathing disorders (SRBDs) including OSA, and CSA in hemodialysis (HD) patients has been established as s significant cardiovascular disease (CVD) risk (Sakura et al., 2016; Walker et al., 1995). Since volume overload and inadequate dialysis cause either OSA or CSA, co‐existence of SRBDs may contribute to the development of CVD events in HD patients (Perl et al., 2006; Tang et al., 2009). Therefore, to examine the association of poor SQ with CVD risk, their association should be investigated after the exclusion of the effect of OSA (Walker et al., 1995 **),** CSA(Sakura et al., 2016), and sleep disorder‐induced impaired metabolisms.

In the present study, we evaluated (i) the correlation of SQ markers including polysomnography (PSG) determined REM‐SL with various metabolic markers at baseline in HD patients and (ii) the association of baseline level of REM‐SL with the development of CVD events in a longitudinal design in HD patients, after excluding the influence of OSA, CSA, and metabolic markers at baseline.

## 2 SUBJECTS AND METHODS

### 2.1 Study participants characteristics

We requested 810 maintenance HD patients who visited the HD outpatient department of Shirasagi Hospital Kidney Center from August 2010 to June 2015 to participate in this study. Of them, 497 HD patients agreed to undergo a 3‐day continuous screening nocturnal oximetry (PULSOX‐Me300, Konica Minolta, Inc.). The criteria for participation in this study were set to oxygen desaturation index: ODI3 ≥ 10 on a 3‐day average. Exclusion criteria were set for patients with acute illness, infection, <1 year HD period, and malignancy. Of the 497 patients with consent, 249 met these criteria. Eventually, 88 of them agreed to participate in the study and underwent PSG. The entry period was from August 2010 to June 2015, and the occurrence of CVD events was followed up and monitored until June 2016. This study was approved by the Ethics Review Committee of Shirasagi Hospital (Approval # 22–0112), and was conducted in accordance with the principles of the Declaration of Helsinki.

Before the PSG study, baseline data such as gender, age, HD duration, dialysis time, body weight, were collected. Pre‐existing CVD disease were determined by clinical chart review and patient survey at the point of enrollment to study. The diagnosis of diabetes mellitus (DM) was made on the basis of a history of DM or according to the Japan Diabetes Society criteria (Seino et al., 2010). Cardiothoracic ratio, systolic blood pressure, and serum samples were collected just before the first morning HD session of the week. Serum laboratory parameters were measured using an auto analyzer (Hitachi 7450; Hitachi Co.). Non‐HDL‐C levels were calculated by subtracting HDL‐C levels from the total cholesterol levels. HbA1c was measured by routine HPLC (High Performance Liquid Chromatography) and latex agglutination immunoassay, and expressed as the National Glycohemoglobin Standardization Program equivalent value (Kashiwagi et al., 2012). To evaluate the adequacy of regular dialysis therapy, calculations of Kt/V‐urea from the data of routine laboratory examinations by means of urea kinetic modeling were performed (Daugirdas, 1993).

These data were obtained from the same approach of our previous report (Sakura et al., 2016).

### 2.2 Measurement and analysis of PSG data

Overnight PSG (EEG‐9200; Nihonkoden Co.) was performed on the same day when HD study participants received HD session, as we reported previously in the Shirasagi Hospital sleep laboratory. Sleep recording was performed by PSG technologist in the hospital. As measures of sleep architecture, total sleep time, REM‐SL, sleep efficiency, and sleep stages were classified by EEG into 4 stages, WASO, stage 1, stage2, slow wave sleep stage, and REM stage. Sleep is a dynamic state with its own distinctive stages that cycle throughout the night. The succession of cycles, their component stages, and the length of each stage and cycle comprise a person's sleep architecture (Dement & Kleitman, 1957). Among them, we and others have shown that REM‐SL, which is defined as an interval between sleep‐onset and the first REM period might provide a clinically relevant objective measure for SQ (Kostic et al., 1989). REM sleep latency means first sleep cycle, the most important deepest sleep cycle. Delta power density (the marker of deep sleep) is highest in this interval during sleep time (Aeschbach & Borbely, 1993). Apnea was defined as a cessation of airflow for at least 10 s and hypopnea was defined as a 50% or greater decrease in the amplitude, resulting in a decrease of at least 3% in the arterial oxyhemoglobin saturation (Berry et al., 2012). Sleep apnea was defined as an AHI >5/h. Apnea was further classified into CSA and OSA when thoracoabdominal motion was absent or present on elctromyogram, respectively. Hypopnea was also classified as central or obstructive in the presence or absence of out‐of‐phase thoracoabdominal motion, respectively (Berry et al., 2012). Mixed apnea was classified as CSA.

### 2.3 Outcome data collection

The HD cohort was followed up to the end of June 2016 with a mean and a median (range) follow‐up period of 32 and 33 (1–64) months, respectively. The occurrence of CVD events was evaluated based on clinical information regarding coronary artery disease and cerebrovascular disease. The occurrence of new CVD events during study were reported from attending doctors assigned to work with Shirasagi Hospital to the Shirasagi Hospital sleep laboratory. CVD was diagnosed when a study participant fulfilled one or more of the following criteria: (1) presence of significant stenosis by coronary angiography, (2) presence of ST‐T abnormalities on an electrocardiogram associated with typical symptoms attributable to angina pectoris, and (3) presence of cerebrovascular disease by CT or MRI with typical symptoms attributable to cerebral stroke.

### 2.4 Statistical analysis

Continuous variables are expressed as the mean ± standard deviation (SD). Medians (interquartile range) were obtained for variables with a non‐normal distribution. Correlation coefficients were calculated using simple and multiple regression analysis after the logarithmic transformation of non‐normally distributed variables. Simple regression analysis was performed using Student's *t*‐test. Kaplan–Meier analysis and log‐rank tests were used to compare the CVD events‐free survival rates between two groups based on REM‐SL. Data of nine study participants died by non‐CVD and 12 study participants moving away from Shirasagi Hospital were censored at the date of the participant's last known disease evaluation. Stepwise logistic regression was used to select the predictors of CVD events. Multivariable Cox proportional hazards regression models were used to determine independent predictors of future CVD events among the same candidate variables. Estimated hazard ratios along with corresponding 95% confidence intervals and P‐values were obtained for all regression covariates. *p*‐values <0.05 were considered statistically significant. All data were analyzed using Stat View version 5.0 J (Abacus Concepts, Inc.). These statistical Analysis were performed by the same way of our previous report (Sakura et al., 2016).

## 3 RESULTS

### 3.1 Baseline clinical profiles and sleep architecture of 88 enrolled HD study participants

The baseline clinical and biochemical profiles of the 88 HD study participants are shown in Table 1. The mean (±SD) age was 68.4 ± 9.3 years, and 63.6% of the study participants were male. The median BMI and dialysis duration were 21.8 (range: 19.5–24.6) kg/m^2^ and 6.8 (range: 3.0–13.0) years, respectively. There were 36 study participants (40.9%) with T2DM and 34 study participants (38.6%) with pre‐existing CVD. The number of HD study participants under treatment with antihypertensive medications, statins, and prescribed sleep medications were 65 (73.8%), 23 (26.1%), and 50(56.8%), respectively. When the study participants analyzed were restricted to those with T2DM (n = 36), mean levels of fasting plasma glucose (FPG) and HbA1c were 151.5 ± 33.4 mg/dL and 6.2 ± 0.8%, respectively. The mean (±SD) Kt/V was 1.6 ± 0.2.

**TABLE 1:** Baseline profiles and sleep architecture of 88 hemodialysis patients

Measures	HD patients (n = 88)
Baseline profiles	
Gender (male/female)	56/32
Age (years)	68.4 ± 9.3
Dialysis duration (years)	6.8 [3.0–13.0]
BMI (Kg/m^2^)	21.8 [19.5–24.6]
Type 2 DM, n (%)	36 (40.9)
Pre‐existing CVD, n (%)	34 (38.6)
Cardiothoracic ratio (%)	49.2 ± 4.8
Systolic blood pressure (mm Hg)	157.8 ± 19.5
Albumin (g/dL)	3.6 ± 0.2
Hemoglobin (g/dL)	10.6 ± 0.8
T‐Chol (mg/dL)	153.7 ± 28.5
TG (mg/dL)	120.1 ± 69.6
HDL‐C (mg/dL)	44.4 ±13.5
Non‐HDL‐C (mg/dL)	109.3 ± 25.9
FPG (mg/dL)	126.5 ± 33.7
Kt/V	1.6 ± 0.2
Sleep architecture	
Total sleep time (min)	349.0 [266.0–404.5]
REM sleep latency (min)	150.5 [92.0–260.5]
Sleep efficiency (%)	56.9 [44.0–64.3]
Sleep stage (min)	
Wake after sleep onset (WASO)	57.0 [24.5–154.5]
Rapid eye movement (REM)	39.0 [22.9–60.6]
Stage 1	106.1 [74.1–143.59]
Stage 2	166.4 [119.9–223.9]
Slow wave sleep (SWS)	0 [0–0.5]
Sleep apnea	
Apnea hypopnea index (AHI) (no./h)	36.0 [21.5–55.4]
Central AHI (no./h)	0.7 [0.2–3.3]
Mean oxygen saturation (%)	95.8 ± 1.7

Data are expressed in mean ± SD or median [interquartile range]

### 3.2 Univariate correlations of the metabolic variables with sleep architecture

**FIGURE 1:**
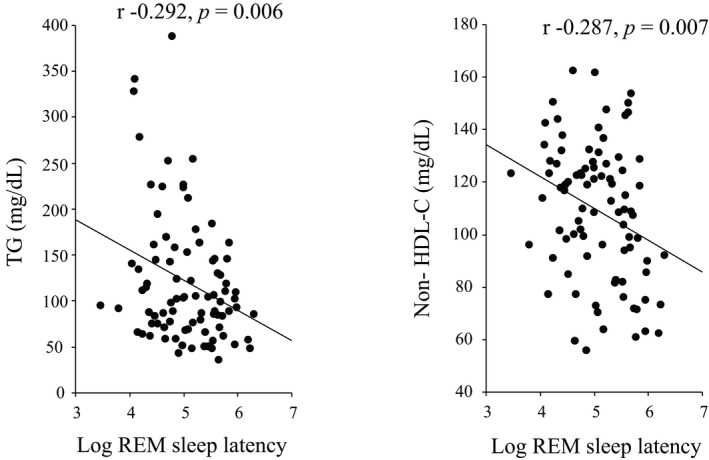
Univariate correlation of TGs and non‐HDL‐c with REM sleep latency in all patients (n = 88)

**FIGURE 2:**
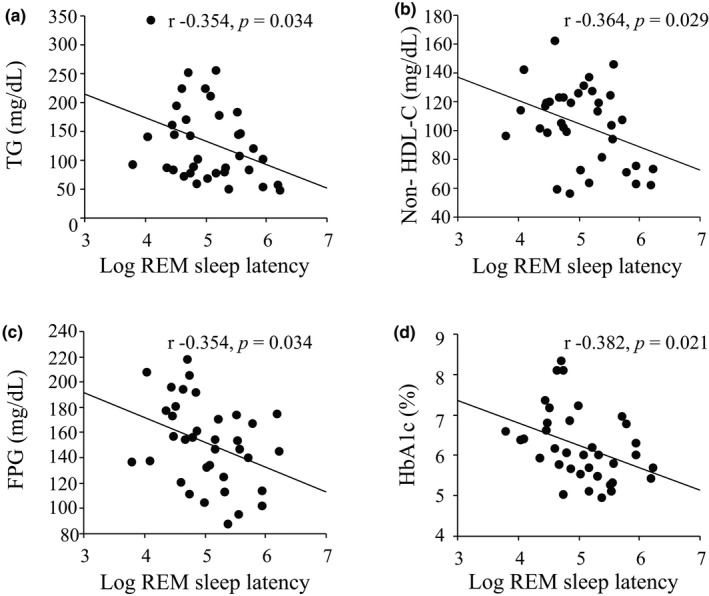
Univariate correlation of TGs, non‐HDL‐c, FPG, and HbA1c with REM sleep latency in diabetes patients (n = 36)

With regards to sleep architecture of the study participants (Table 1), total sleep time, REM‐SL, and sleep efficiency were 349.0 (266.0–404.5) min, 150.5 (92.0–260.5) min, and 56.9 (44.0–64.3) %, respectively. Slow wave sleep was almost deficient, possibly due to the old age (68.4 years) of the enrolled study participants. Since accumulating evidence including ours (Yoda et al., 2015) indicate that SQ is intimately associated with various metabolic disorders such as lipid profiles, and glycemic control in DM patients (Bruehl et al., 2007; Poland et al., 1992), we next examined the univariate correlations of various metabolic variables with various parameters of SQ after logarithmical transformation of sleep parameters to make them normally distributed (Supplement 1), and examined the univariate correlations of various clinical variables with REM‐SL (Table 2). We found that REM‐SL alone might provide a clinically relevant measure for SQ, based on its significant and negative correlations with triglycerides (TGs) (r = −0.292, *p* = 0.006) and non‐HDL‐C (r = −0.287, *p* = 0.007) in all study participants (Figure 1) and with FPG (r = −0.354, *p* = 0.034) and HbA1c (r = −0.382, *p* = 0.021) in 36 T2DM HD study participants (Figure 2). These results are consistent with our previous report indicating that REM‐SL is a clinically useful marker of poor SQ that exacerbates lipid and glucose metabolism (Yoda et al., 2015). Furthermore, univariate analysis to identify the association of parameters of sleep architecture with new‐onset CVD events showed log (REM‐SL) alone, but nor other parameters, as significant factor associated with new‐onset CVD events (HR 0.717 [95% CI: 0.540–0.953] (Table 3). Thereafter, we adopted REM‐SL as an objective measure of SQ to examine the association of impaired SQ with CVD events in HD study participants.

**TABLE 2:** Univariate correlations of various clinical variables with REM sleep latency

Measure	Log REM sleep latency		
	r	*p*	95%CI
Gender (male = 1/female = 2)	−0.036	0.744	−0.321–0.230
Age (years)	0.020	0.856	−0.013–0.015
Log Dialysis duration	0.009	0.933	−0.122–0.133
Log BMI	0.143	0.185	−1.320–0.259
Cardiothoracic ratio (%)	0.109	0.316	−0.013–0.041
Systolic blood pressure (mm Hg)	0.035	0.747	−0.006–0.008
Log Apnea hypopnea index	0.137	0.204	−0.070–0.319
Log (Central apnea hypopnea index+1)	−0.039	0.717	−0.158–0.109
Mean oxygen saturation	−1.111	0.306	−0.118–0.037
Hemoglobin (g/dL)	−0.092	0.394	−0.229–0.091
Albumin (g/dL)	0.081	0.456	−0.279–0.656
TG (mg/dL)	−0.292	0.006*	−0.004–−0.001
LDL‐C (mg/dL)	−0.141	0.191	−0.009–0.002
HDL‐C (mg/dL)	0.096	0.377	−0.005–0.014
Non‐HDL‐C (mg/dL)	−0.287	0.007*	−0.012–−0.002
FPG (mg/dL)	−0.159	0.141	−0.007–0.001
Kt/V	0.047	0.668	−0.069–0.108
Diabetes mellitus (n = 36)			
FPG (mg/dL)	−0.354	0.034*	−4.990–−0.012
HbA1c (%)	−0.382	0.021*	−0.458–−0.041

* *p* < 0.05

**TABLE 3:** Univariate Cox proportional hazard analysis of the CVD incidence.

Measure	Hazard Ratio^a^	95%, C.I.^a^	*p* value
Log Total sleep time	1.081	0.691–1.692	0.732
Log REM sleep latency	0.717	0.540–0.953	0.022*
Log Sleep efficiency	0.992	0.645–1.526	0.971
Sleep stage time			
Log WASO	1.107	0.722–1.698	0.640
Log Stage 1	0.951	0.612–1.478	0.822
Log Stage 2	1.274	0.704–2.307	0.484
Log Slow wave sleep	0.755	0.430–1.326	0.328
Log REM	0.902	0.595–1.366	0.569
Log Apnea hypopnea index	0.988	0.510–1.912	0.971
Log (Central apnea hypopnea index+1)	1.029	0.987–1.072	0.176
Mean oxygen saturation	1.026	0.790–1.331	0.848

^a^ Per 1SD

* *p* < 0.05

### 3.3 Kaplan–Meier analysis and multivariate analysis using Cox proportional hazards models to evaluate the significance of REM‐SL as a risk factor for the development of a new‐onset CVD events in HD study participants

At the end of the follow‐up, 46 study participants were free of CVD events and 21 study participants developed new CVD events. The remaining 21 study participants were censored during the follow‐up. Nine study participants died by non‐CVD and 12 study participants moving away from Shirasagi Hospital. CVD events were found in 11 study participants as significant stenosis by coronary angiography plus typical symptoms attributable to coronary artery disease, 4 study participants as ST‐T abnormalities on an electrocardiogram associated with typical symptoms attributable to angina pectoris, and 6 study participants as CVD disease by CT or MRI with typical symptoms attributable to cerebral stroke.

**FIGURE 3:**
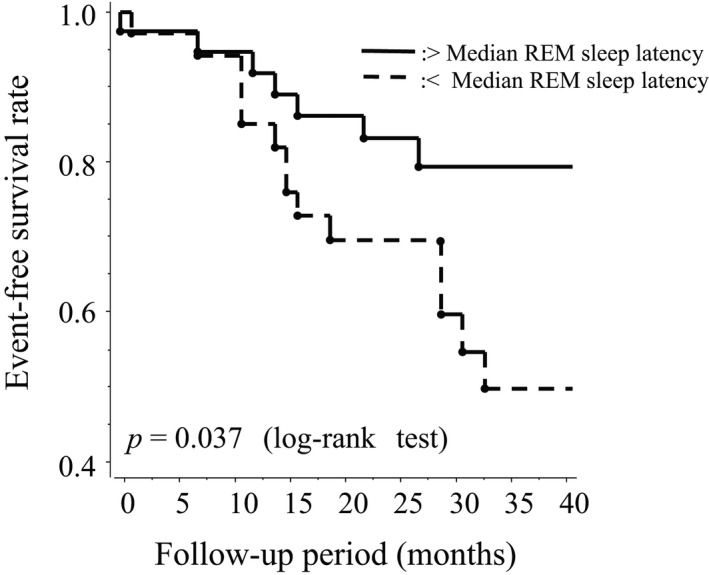
Kaplan–Meier CVD Event‐free survival curves with REM sleep latency above and below the group median

A Kaplan–Meier analysis was performed to examine the univariate association between REM‐SL and new‐onset CVD events in HD study participants. After separating the 88 HD study participants into two equal groups of study participants based on the median value of REM‐SL, it was shown that HD study participants with lower REM‐SL had a significantly lower rate of new‐onset CVD events than those with higher REM‐SL (*p* = 0.037, log‐rank test) (Figure 3).

Next, a univariate Cox proportional hazard analysis of the CVD incidence was performed (Table 3), and then a stepwise logistic regression analysis to identify risk factors for new‐onset CVD events. Thirteen items were included as independent variables comprising basic factors (age, gender, and log [dialysis duration]), CVD‐related factors (pre‐existing CVD, systolic blood pressure, non‐HDL‐C, Statins use and Diabetes mellitus), and sleep‐related factors ([log AHI, log (central AHI+1), mean oxygen saturation and sleep‐promoting drug use). Of these, age, gender, log (dialysis duration), and log (REM‐SL) retained statistical significance as independent factors for new onset CVD events (Table 4).

**TABLE 4:** Factors influencing CVD event onset using by stepwise logistic regression analyses

Step	Variable		Score Χ^2^	*p*
	Entered	Removed		
1	Age		7.272	0.007*
2	Gender		5.457	0.019*
3	Log REM sleep latency		5.069	0.024*
4	Log Dialysis duration		3.912	0.047*
5		Diabetes mellitus	3.690	0.054
6		Cardiovascular disease history	3.622	0.057
7		Log (Central apnea hypopnea index+1)	3.388	0.065
8		Mean oxygen saturation	1.090	0.296
9		Sleep‐promoting drug use	0.967	0.325
10		Systolic blood pressure	0.298	0.585
11		non‐HDL‐Cho	0.255	0.613
12		Statins use	0.189	0.663
13		Log Apnea hypopnea index	0.069	0.793

Basic factors:Gender, Age, Dialysis duration.

CVD‐related factors:Diabetes mellitus, Systolic blood pressure, non‐HDL‐Cho, Statins use, Cardiovascular disease history.

Sleep related factors:REM sleep latency, Apnea hypopnea index, Central apnea hypopnea index, Mean oxygen saturation, Sleep‐promoting drug use.

* *p* < 0.05.

To confirm that the independent association of log (REM‐SL) as well as age, gender, and log (dialysis duration), with new‐onset CVD events is not affected by lipid metabolism or CSA, multivariate Cox proportional hazards regression analysis was performed (Table 5). In Model 1, which included log (REM‐SL), age, gender, and log (dialysis duration), each parameter was significantly associated with new‐onset CVD events in HD study participants. Model 2, 3, 4, 5, 6 and 7 in which non‐HDL‐C, log (central AHI+1), CVD history, Diabetes mellitus and Systolic blood pressure respectively, were added to Model 1, showed that log (REM‐SL) remained a significant and independent risk factor for a new‐onset CVD events.

**TABLE 5:** Multivariable Cox proportional hazard regression models for CVD event onset (n = 88)

	Model 1			Model 2			Model 3		
	HR	95% CI	*p*	HR	95% CI	*p*	HR	95% CI	*p*
Age (1 SD)	2.058	1.218–3.478	0.007*	1.993	1.161–3.422	0.012*	2.120	1.250–3.594	0.005*
Gender (M = 1, F = 2)	0.256	0.082–0.803	0.019*	0.275	0.085–0.892	0.031*	0.238	0.076–0.752	0.014*
Log Dialysis duration (1 SD)	1.552	1.004–2.400	0.047*	1.573	1.011–2.446	0.044*	1.574	1.009–2.453	0.045*
Log REM sleep latency (1 SD)	0.503	0.277–0.915	0.024*	0.483	0.261–0.893	0.020*	0.519	0.281–0.959	0.036*
non‐HDL‐Cho (1 SD)				0.869	0.503–1.501	0.613			
Log(Central apnea hypopnea index+1) (1 SD)							1.411	0.969–2.054	0.072
Mean oxygen saturation (1 SD)									
Cardiovascular disease history(non = 0, yes = 1)									
Diabetes mellitus (non = 0, yes = 1)									
Systolic blood pressure (1 SD)									

	Model 4			Model 5			Model 6		
	HR	95% CI	*p*	HR	95% CI	*p*	HR	95% CI	*p*
Age (1 SD)	2.126	1.259–3.589	0.004*	1.993	1.168–3.400	0.011*	2.281	1.304–3.990	0.003*
Gender (M = 1, F = 2)	0.227	0.071–0.725	0.012*	0.245	0.077–0.786	0.018*	0.282	0.089–0.898	0.032*
Log Dialysis duration (1 SD)	1.067	1.004–1.135	0.037*	1.629	1.053–2.519	0.028*	1.746	1.123–2.715	0.013*
Log REM sleep latency (1 SD)	0.498	0.275–0.899	0.020*	0.508	0.288–0.894	0.018*	0.523	0.286–0.954	0.034*
non‐HDL‐Cho (1 SD)									
Log(Central apnea hypopnea index+1) (1 SD)									
Mean oxygen saturation (1 SD)	0.753	0.440–1.286	0.298						
Cardiovascular disease history(non = 0, yes = 1)				2.321	0.953–5.654	0.063			
Diabetes mellitus (non = 0, yes = 1)							2.442	0.964–6.187	0.059
Systolic blood pressure (1 SD)									

	Model 7		
	HR	95% CI	*p*
Age (1 SD)	2.109	1.234–3.605	0.006*
Gender (M = 1, F = 2)	0.264	0.084–0.827	0.022*
Log Dialysis duration (1 SD)	1.569	1.013–2.429	0.043*
Log REM sleep latency (1 SD)	0.492	0.270–0.899	0.021*
non‐HDL‐Cho (1 SD)			
Log(Central apnea hypopnea index+1) (1 SD)			
Mean oxygen saturation (1 SD)			
Cardiovascular disease history(non = 0, yes = 1)			
Diabetes mellitus (non = 0, yes = 1)			
Systolic blood pressure (1 SD)	1.140	0.713–1.822	0.585

Abbreviation: HR, Hazards ratio.

* *p* < 0.05

## 4 DISCUSSION

The present study demonstrated that study participants with shorter REM‐SL, which is defined as an interval between sleep‐onset and the first REM period and as a measure of the most important deep sleep during the first sleep cycle (Geyer et al., 2009) had a significantly higher risk for new‐onset CVD events than those with longer REM‐SL independent of AHI and central AHI (Figure 3 &Table 5). Although REM‐SL was correlated with clinical parameter related to lipid metabolism in all study patients and glucose metabolism in T2DM study participants. Multivariate analysis showed that REM REM‐SL retained its significant association with new‐onset CVD events even when non‐HDL‐C was included as an independent variable, clearly suggesting poorer SQ as a significant CVD risk factor in HD patients, independent of obstructive and central apnea, or metabolic factors.

When REM‐SL, as well as other sleep markers including sleep time and sleep stages, was examined for its correlation with TGs and non‐HDLC in all study participants, and with FPG and HbA1c in T2DM HD study participants (Supplement 1), we found that REM‐SL correlated significantly with these metabolic markers in HD study participants, justifying the adoption of REM‐SL as a marker for SQ in the present study. Further, supportive of this notion that log (REM SL) alone, but not other parameters, was significant factor associated with new‐onset CVD events. We have reported previously in a cross‐sectional study that shorter REM‐SL, but not other measurements of SQ thus far reported, is associated with higher intima‐media thickness at common carotid artery (CA‐IMT) in T2DM patients (Yoda et al., 2015). The results of the current study further confirmed the notion that impaired SQ is a treatment target to protect against the development of CVD events in HD patients, based on an independent association of shorter REM‐SL with higher risk of new onset CVD events.

However, since there is accumulating evidence to indicate that poor SQ is a significant factor to induce metabolic disorders, such as abnormalities in lipid and glucose metabolism, obesity, non‐dipper hypertension (Hamamoto et al., 2016; Javaheri & Redline, 2012), and morning hypertension (Yoda et al., 2015) to exacerbate atherosclerotic changes, it is important to know whether or not the association of SQ with new‐onset CVD events might be confounded by metabolic abnormalities.

Furthermore, it is known that poorer SQ might be significant factor to inducing non dipping hypertension, morning hypertension, and exacerbation of atherosclerotic changes (Javaheri & Redline, 2012) possibly due to sympathomimetic effects (Campbell et al., 1985). We have previously raised the possibility that impaired SQ due to higher disease activity of rheumatoid arthritis might play a role in the development of non‐dipper hypertension (Hamamoto et al., 2016). Taken these data into consideration, although stepwise logistic regression analysis (Table 4) was performed, analysis negated the association of systolic blood pressure, non‐HDL‐C, and DM with new‐onset CVD events. Even multivariate Cox proportional hazard regression analysis (Table 5) demonstrated the lack of the effect on the association of REM‐SL with new‐onset CVD events.

Furthermore, it has been established that OSA and CSA occur frequently in HD patients, and that either OSA or CSA are independent factors of CVD events in HD patients (Masuda et al., 2011; Rod et al., 2011). Sleep‐disordered breathing has been associated not only with poor SQ but also with hypertension and increased risk of CVD (Shaw et al., 2008). It has been shown that the physiologic stress imposed by intermittent hypoxia and/or sleep fragmentation may cause sympathetic activation with catecholamine elevation, cortisol elevation, oxidative stress, and activation of inflammation (Shaw et al., 2008) However, even after exclusion of the effect of OSA or CSA (Table 2&3), log (REM‐SL) still retained its significant association with new‐onset‐CVD events, suggesting that poorer SQ may be a clinically significant risk factor on CVD risk in HD patients, independent of its metabolic effect, OSA, or CSA.

An association of nocturnal hypoxemia with mortality in patients with advanced kidney disease and dialysis patients has been reported (Manisha et al., 2020). The reason why no association was found in this study may be that the mean (SD) oxygen saturation was 95.8 (1.7) in our study, which was higher than that in the previous studies.

This study with one facility has several limitations. Firstly, it was a small‐scale study. Secondly, since the present study enrolled only Japanese HD patients, getting treatment under the same policy in HD Center of Shirasagi Hospital, it is uncertain whether the results demonstrated in our study applied to other ethnicities. Thus, the results demonstrated from our study should be verified in a larger‐scale study and also in other ethnicities. Other possible limitations include the issue of selection bias. More health‐conscious patients and those who can spend more time on inpatient examinations may have agreed to the study more than others.

In summary, the present study investigated the role of objectively measured SQ beside normal know risk factors over CVD events in HD patients. Eighty‐eight subjects undergoing HD were evaluated and follow up for median 33 months after a PSG. Among objectively measured SQ, a reduction of REM‐SL was associated with poor lipid control in HD patients and with poor glycemic control in type‐2 diabetic HD study participants. The decrease of REM‐SL observed in this sleep study increased the rate of new onset CVD events independently of SRBDs (OSA and CSA), lipid control and DM. Therefore, the present study suggests REM‐SL might be a novel risk factor of new onset CVD events in HD patients with SRBDs.

## CONFLICTS OF INTEREST

We declare that we have no conflicts of interest to disclose.

## Supporting information



Supplementary Material
