# Supplementary material for: REM sleep latency as an independent risk for cardiovascular events in hemodialysis patients
Source: Physiol Rep. 2021 May 15;9(9):e14837. doi: 10.14814/phy2.14837 (PMC8123536; doi:10.14814/phy2.14837)
Supplement: Supplementary file 1 — Supplementary Material [file PHY2-9-e14837-s001.pptx]

## Slide 1
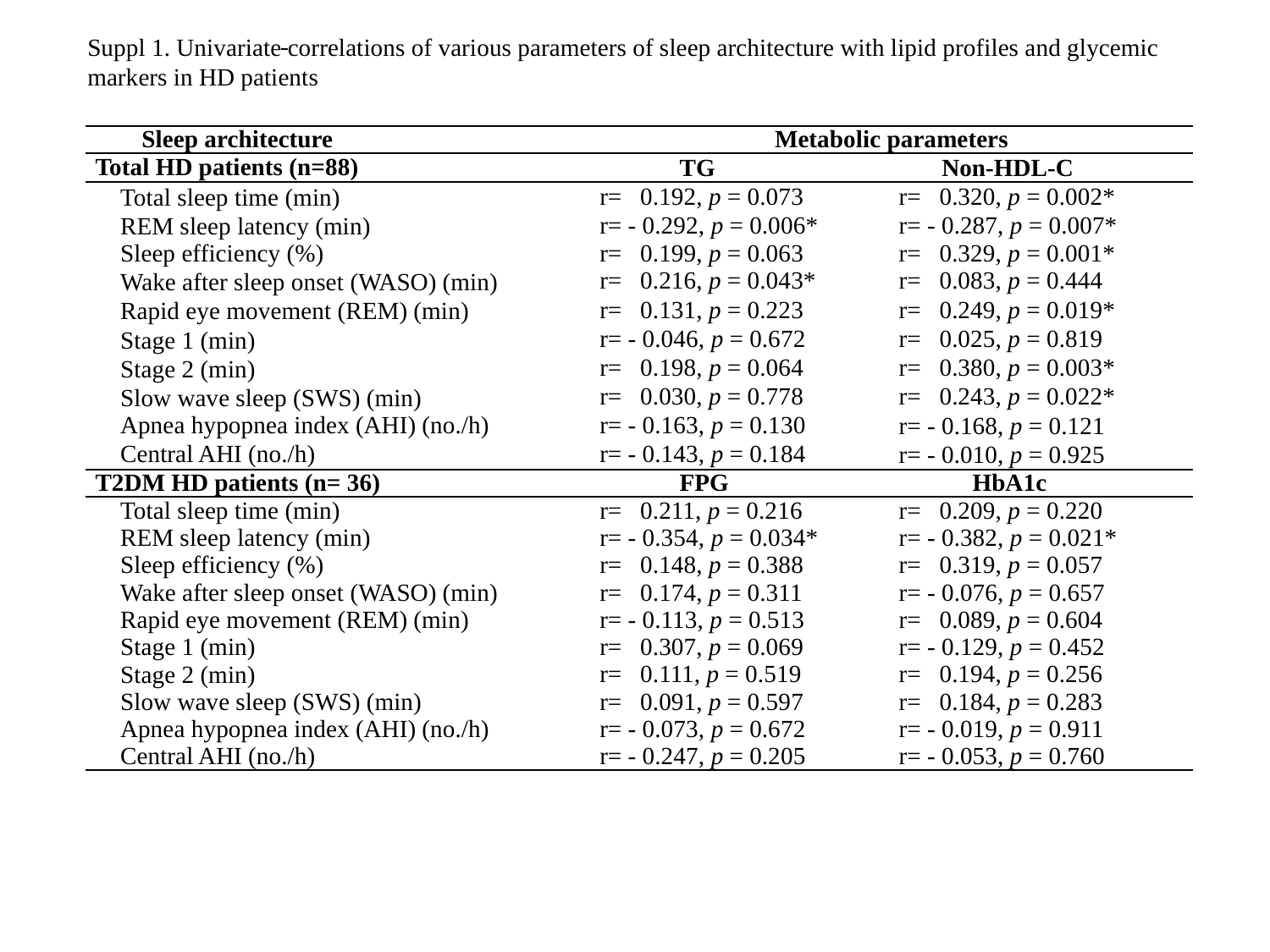

Suppl 1. Univariate correlations of various parameters of sleep architecture with lipid profiles and glycemic markers in HD patients
| Sleep architecture | Metabolic parameters | |
| --- | --- | --- |
| Total HD patients (n=88) | TG | Non-HDL-C |
| Total sleep time (min) | r= 0.192, p = 0.073 | r= 0.320, p = 0.002\* |
| REM sleep latency (min) | r= - 0.292, p = 0.006\* | r= - 0.287, p = 0.007\* |
| Sleep efficiency (%) | r= 0.199, p = 0.063 | r= 0.329, p = 0.001\* |
| Wake after sleep onset (WASO) (min) | r= 0.216, p = 0.043\* | r= 0.083, p = 0.444 |
| Rapid eye movement (REM) (min) | r= 0.131, p = 0.223 | r= 0.249, p = 0.019\* |
| Stage 1 (min) | r= ‐ 0.046, p = 0.672 | r= 0.025, p = 0.819 |
| Stage 2 (min) | r= 0.198, p = 0.064 | r= 0.380, p = 0.003\* |
| Slow wave sleep (SWS) (min) | r= 0.030, p = 0.778 | r= 0.243, p = 0.022\* |
| Apnea hypopnea index (AHI) (no./h) | r= - 0.163, p = 0.130 | r= - 0.168, p = 0.121 |
| Central AHI (no./h) | r= - 0.143, p = 0.184 | r= - 0.010, p = 0.925 |
| T2DM HD patients (n= 36) | FPG | HbA1c |
| Total sleep time (min) | r= 0.211, p = 0.216 | r= 0.209, p = 0.220 |
| REM sleep latency (min) | r= ‐ 0.354, p = 0.034\* | r= - 0.382, p = 0.021\* |
| Sleep efficiency (%) | r= 0.148, p = 0.388 | r= 0.319, p = 0.057 |
| Wake after sleep onset (WASO) (min) | r= 0.174, p = 0.311 | r= - 0.076, p = 0.657 |
| Rapid eye movement (REM) (min) | r= ‐ 0.113, p = 0.513 | r= 0.089, p = 0.604 |
| Stage 1 (min) | r= 0.307, p = 0.069 | r= - 0.129, p = 0.452 |
| Stage 2 (min) | r= 0.111, p = 0.519 | r= 0.194, p = 0.256 |
| Slow wave sleep (SWS) (min) | r= 0.091, p = 0.597 | r= 0.184, p = 0.283 |
| Apnea hypopnea index (AHI) (no./h) | r= - 0.073, p = 0.672 | r= - 0.019, p = 0.911 |
| Central AHI (no./h) | r= - 0.247, p = 0.205 | r= - 0.053, p = 0.760 |
